# In-Air Electron Streaming Effect for Esophageal Cancer Radiotherapy With a 1.5 T Perpendicular Magnetic Field: A Treatment Planning Study

**DOI:** 10.3389/fonc.2020.607061

**Published:** 2020-12-01

**Authors:** Hongdong Liu, Shouliang Ding, Bin Wang, Yongbao Li, Ying Sun, Xiaoyan Huang

**Affiliations:** State Key Laboratory of Oncology in South China, Collaborative Innovation Center for Cancer Medicine, Department of Radiation Oncology, Sun Yat-sen University Cancer Center, Guangzhou, China

**Keywords:** electron streaming effect, MR-linac, MR guided radiotherapy, esophageal cancer, out-of-field dose

## Abstract

**Purpose:**

To investigate the in-air out-of-field electron streaming effect (ESE) for esophageal cancer radiotherapy in the presence of 1.5 T perpendicular magnetic field.

**Methods:**

Ten esophageal cancer patients treated with conventional Linac were retrospectively enrolled into a cohort of this study, with the prescription of 4,400 cGy/20 fx. All cases received IMRT replanning using Elekta Unity MR-Linac specified Monaco system, denoted as primary plan. To visualize the in-air dose outside the body in Monaco system, an auxiliary structure was created by extending the external structure. For each case, another comparable plan with no magnetic field was created using the same planning parameters. The plan was also recalculated by placing a bolus upon the neck and chin area to investigate its shielding effect for ESE. Dosimetric evaluations of the out-of-field neck and chin skin area and statistical analysis for these plans were then performed.

**Results:**

Out-of-field ESE was also observed in esophageal cancer treatment planning under 1.5 T magnetic field, while totally absent for plans with no magnetic field. On average, the maximum dose to the neck and chin skin area of the primary plan (657.92 ± 69.07 cGy) was higher than that of plan with no magnetic field (281.78 ± 36.59 cGy, p = 0.005) and plan with bolus (398.43 ± 69.19 cGy, p = 0.007). DVH metrics D_1cc_ (the minimum dose to 1 cc volume) of the neck and chin skin for primary plan was 382.06 ± 44.14 cGy, which can be reduced to 212.42 ± 23.65 cGy by using the 1 cm bolus (with p = 0.005), even lower than the plan without magnetic field (214.45 ± 23.82, p = 0.005). No statistically significant difference of the neck and chin skin dose between the plan with bolus and plan with no magnetic field was observed (all with p > 0.05).

**Conclusion:**

For MRI guided esophageal cancer radiotherapy, a relatively high out-of-field neck and chin skin doses will be introduced by ESE in the presence of magnetic field. It is therefore recommended to take this into account during the planning phase. Adding bolus could effectively reduce the ESE dose contributions, achieve the shielding effect almost equivalent to the scenario with no magnetic field. Further explorations of measurement verifications for the ESE dose distributions are required.

## Introduction

Esophageal cancer is the seventh most commonly occurring cancer worldwide, with more than half million new cases diagnosed in 2018 (1). According to the statistic, new cases occur more commonly in the less developed countries and in males (1, 2). Esophageal cancer is highly lethal. Although surgery is currently the mainstay approach, radiation therapy also plays a critical role in the treatment of esophageal cancer. Image-guided radiotherapy (IGRT) with cone-beam computed tomography (CBCT) is commonly used in practice for patient set-up verification in esophageal cancer treatment. But inter and intra fraction tumor variations are still a dominant uncertainty in esophageal cancer radiotherapy, such as target deformation and respiration motion, which are generally not possible to be solved by conventional IGRT with CBCT (3).

In recent years, MRI-guided radiotherapy (MRIgRT) system has undergone substantial development and become clinically available with the implementation of the MR-Linac (MRL) device (4, 5). Esophageal cancer patients are considered to potentially benefit from better guidance in the MRL (6, 7). MRI can yield a superior soft-tissue contrast when compared with CBCT, which not only means that both the tumor and surrounding organs at risk (OARs) can be recognized more clearly, but also make it possible for soft tissue based set-up correction (7). Moreover, MRL is able to capture both the inter and intra fraction motion and anatomic changes, making it possible for real-time tumor tracking and adaptive replanning (8–12). MRL system is thereby considered suitable for esophageal cancer radiotherapy, which potentially allows a reduced target margin, better dose conformity, target dose escalation and less toxicity to the OARs, *etc* (6, 7). In our institute, the Elekta Unity MRL system (Elekta AB, Stockholm, Sweden) has been introduced and installed recently. This system consists of a Phillips Ingenia 1.5 T MR scanner (Best, The Netherlands), surrounded by a ring-shaped gantry on which a 7 MV linear accelerator has been mounted (13, 14). The magnetic field orientation points out the device bore entrance and is always perpendicular to the 7 MV radiation beam.

The presence of perpendicular magnetic field of the MRL system can influence the trajectories of secondary electrons generated by photon interacting with the material, thereby changing the dose deposition. Secondary electrons generated inside the patient’s body can be accurately calculated by MRL specific treatment planning system (TPS), and the induced dose variation can be visualized properly (15). Those electrons scattered outside the patient’s body, however, can easily be ignored in that the outside in-air doses are typically not visualized in TPS. In reality, the generated electrons in the air will be focused by the magnetic field, then converged to form electron streams, moving toward the poles of the magnetic field (16, 17). These in-air electrons can drift along the magnetic field line for a distance until they impact a surface, thus having the chance to strike the patient’s body and contribute to skin toxicity outside the treatment field. This electron streaming effect (ESE) is unique for MRL and currently gaining more and more attention. Hackett et al. measured the in-air contaminant electron doses under a perpendicular magnetic field using EBT3 film and reported that the dose to a surface perpendicular to the magnetic field 5 cm outside the field edge is 5.6 ± 0.2% of the maximum dose on the central axis (16). This finding was subsequently confirmed by Malkov et al. using the EGSnrc Monte Carlo simulations (17). Furthermore, Malkov et al. implemented Monte Carlo simulations to study the ESE induced out-of-field surface dose enhancement with oblique phantom under various magnetic field strength, and they found that ESE can produce doses as high as 39.0 ± 0.2% of the maximum deliverable dose by the photon beam (18). An et al. calculated and measured the air-electron stream doses outside the treatment field with ViewRay system by utilizing a custom-made acrylic phantom (19). As to the patient cases, Park et al. investigated the out-of-field ESE with treatment plans and *in-vivo* measurements for accelerated partial breast irradiations on the 0.35 T ViewRay ^60^Co system (20). Nachbar et al. studied the ESE for the first partial breast irradiation patient treated on Elekta Unity 1.5 T MRL, a fractional dose of 0.17 Gy in the chin area (prescription of 2.67 Gy/fx) was reported (21). Such high doses indicate that the generated in-air electron streams are indeed capable of striking the patient body and contributing to skin doses for certain treatment site.

ESE is not merely limited to breast cancer, but ubiquitous for MRL and the strength should be dependent on the treatment site and target location. Such as the esophageal cancer, whose irradiated target volume is typically close to the neck and chin areas, ESE induced skin dose enhancement and toxicity should be higher and thereby need more concerns. The purpose of this work is to investigate the out-of-field ESE for esophageal cancer cases with treatment planning under 1.5 T perpendicular magnetic field. Dosimetric comparisons and analysis were performed as well as the shielding method to minimize the impact, which was then evaluated.

## Material and Methods

### Case Inclusion

Ten patients with diagnosis of esophageal carcinoma were retrospectively selected from our institution’s database and then imported to Unity specified TPS Monaco system (V5.40.1). All the patients were previously treated with IMRT technique on a conventional linac. In our study, patients were selected mainly concerning the systematic limitation of field size. The maximum radiation field size at the isocenter of the Unity MR-linac system is 22 and 57 cm in the craniocaudal (CC) and lateral directions, respectively (22, 23). Another key characteristic of Unity system is the fixed height couch, and set-up errors are typically corrected by adapting the beam apertures (23, 24). A 1 cm isotropic margin was recommended to adapt the daily anatomy and set-up errors (22). This indicates a maximum field length of 20 cm in the CC direction. As a result, the median length of the primary tumor in CC direction of these included patients is 17.5 cm, ranging from 15 to 18.5 cm. The CC target length is defined as the absolute distance between the most cranial and caudal slices of the PTV region.

### Simulation and Contouring

Patients were immobilized with a custom-made cushion and scanned in the supine position using a Philips Brilliance big bore CT scanner (Philips Medical, Cleveland, OH), with the energy of 140 kVp and reconstructed field of view (FOV) of 60 cm. Uniform slice thickness of 0.3 cm was adopted.

All the structures were contoured in the planning CT. The gross tumor volume (GTV) and the involved lymphadenopathy (GTV_nd_) were delineated by a certified radiation oncologist and subsequently reviewed by another senior radiation oncologist specialized in esophageal cancer. Any disagreement was resolved by discussion, and consensus was finally achieved. CTV was created using a margin 0.5 cm around the GTV and GTV_nd_ in the transversal direction (excluding the heart, large vessels, trachea, bronchial tree and lungs), 3 cm in cranial and caudal direction. For planning target volume (PTV), PGTV was defined as an isotropic margin of 0.5 cm added to the GTV combined with GTV_nd_, intended for receiving a dose of 4,400 cGy. Another isotropic margin of 0.5 cm was added on CTV to generate the PCTV, with intended dose of 4,000cGy. Normal tissues including bilateral lungs, heart, and spinal cord were delineated according to our institutional guidelines. According to previously published studies about breast cases, ESE mainly has impact on the out-of-field skin doses (20, 21). An auxiliary structure was therefore created by expanding the external body in anterior for 8 cm in order to fully display the in-air out-of-field dose distributions.

### Treatment Planning and Evaluation

**Figure 1:**
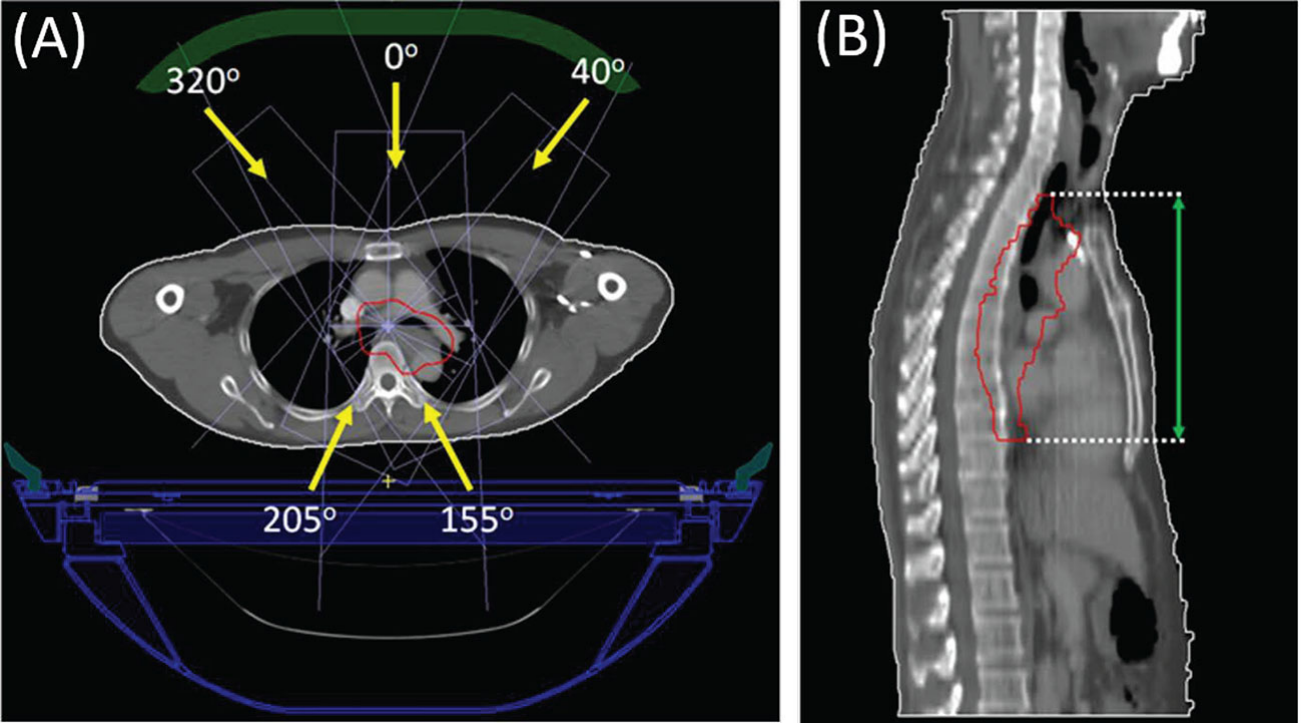
Beam arrangement (subfigure **A**) of a representative case and the target CC length measurement (subfigure **B**) on the sagittal view.

All cases share the same prescription, with the dose of 4,400 Gy in 20 fractions. Simulated plans for all the cases were designed using Unity system dedicated offline treatment planning system (TPS) Monaco (v5.40.01), allowing for plan optimization and dose calculation with consideration of the 1.5 T magnetic field (15). Commissioned Unity machine model was implemented, which is characterized with a nominal beam energy of 7 MV, fixed isocenter and source-to-axis distance (SAD) of 143.5cm. Unity specific couch and coil were also incorporated in the planning; their attenuation to the treatment beam was accounted in the Monaco system. For each case, IMRT plan with step-and-shoot technique was created using a single-isocenter and five coplanar fields, with the gantry angles of 0, 40, 155, 205, and 320°. Due to the existence of a superconducting wire centered within the beam at a gantry angle of 13°, angles ranging from 9 to 17° are therefore avoided (13). **Figure 1** displayed the beam arrangement of one representative case and its target CC length measured in sagittal view. PTV region is depicted in red line, and the green arrow in **subfigure (B)** represents the measuring of target CC length.

Monaco default IMRT parameters allowing up to 250 segments with at least 4 MUs were used for each plan. The minimum segment area and width were set with 2 cm^2^ and 0.5 cm respectively. Plan optimization started with a predefined set of objectives and constraints optimized individually to achieve PTV coverage while minimizing OAR irradiation. **Table 1** listed the predefined planning objectives and constraints used for each case. All plans were optimized to fulfill the dose-volumetric constraints based on our institutional guidelines. Each plan was normalized to cover 95% of the PTV with 100% of the prescription dose. The presence of 1.5 T magnetic field was taken into account throughout the plan optimization and dose calculation process. All the dose calculations were performed with “dose-to-medium” mode by using Monaco’s GPU based Monte Carlo platform GPUMCD (15), with a statistical uncertainty of 1% per control point and a dose grid resolution of 0.1 cm.

**Table 1:** Dose volumetric objectives and constrains for target and OARs.

Structures	Objectives
PGTV	V_44 Gy_ > 95%
PCTV	V_40 Gy_ > 95%
Lungs	V_5 Gy_ < 65%
	V_20 Gy_ < 30%
	V_30Gy_ < 20% D_mean_ < 17 Gy
Spinal cord	D_max_ < 40 Gy
Heart	V_30Gy_ < 30%
	D_mean_ < 26 Gy

According to previous studies, ESE mainly affects the out-of-field in-air dose distribution in CC direction (18–21). For esophageal cancer case, due to the proximity to target, out-of-field neck and chin skin are most likely to be influenced by ESE. In this study, the first 0.5 cm tissue under the external body structure was defined as the skin area. The neck and chin skin dose and dose-volume histogram (DVH) parameters are dependent on how far cranially from the PTV border to the neck skin contour started. For the patient cohort, their PTV to neck skin distances are ranging from 2.7 to 7.5 cm, with the median of 3.9 cm. Dose distribution and DVH metrics for the skin region were then evaluated. To distinguish the ESE induced out-of-field dose variation due to the magnetic field, another comparable plan without the 1.5 T magnetic field was created in the Monaco system, only recalculating the dose and keeping the same planning parameters. Furthermore, the shielding approach was also investigated by virtually placing a 1 cm bolus (with relative electronic density of 1.0) upon the patient’s out-of-field neck or chin area. Dose recalculation was then performed, but with consideration of the virtual bolus. As a result, for each patient there are three plans generated, denoted as primary plan, plan with no magnetic field, and plan with bolus. Out-of-field neck and chin skin doses and DVH parameters between the three plans were compared and analyzed.

Wilcoxon signed rank tests were performed to analyze the skin DVH metric differences between the primary plan, plan with no magnetic field and plan with bolus, using IBM SPSS (v25) software (IBM Corporation, Armonk, NY, USA). Value p < 0.05 is considered statistically significant.

## Results

**Figure 2:**
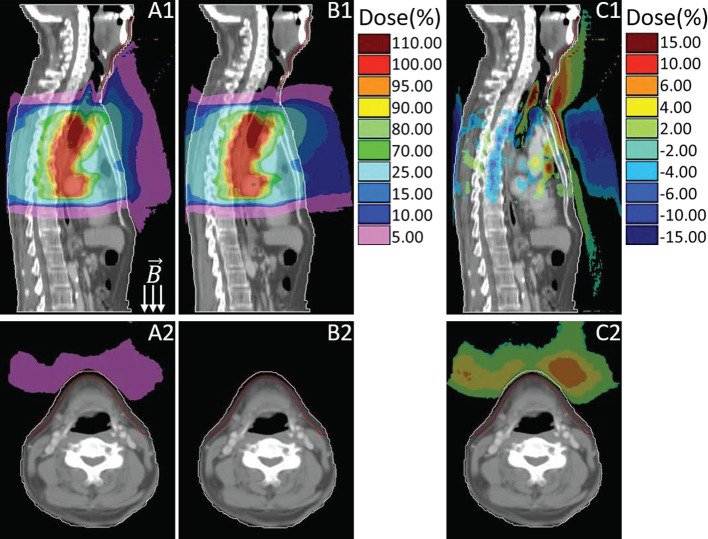
Sagittal and transversal views of the dose distributions for primary plan with magnetic field **(A)**, plan with no magnetic field **(B)** and their dose difference (**C**, primary plan–plan with no magnetic field). Colors: White line delineates the body external, red line represents the neck and chin skin region.

Out-of-field doses were considerably increased due to the presence of ESE under 1.5 T magnetic field. **Figure 2** presents an example of the dose distributions of the aforementioned three plans (primary, no magnetic field, with bolus), all from the same representative patient, and both the sagittal and transversal slice views are provided. In **Figure 2**, **subfigures A1–A2** belong to the primary plan with 1.5 T magnetic field, **B1–B2** are from the plan with no magnetic field, and **C1–C2** are the dose difference by subtracting the plan with no magnetic field from the primary plan. The first row in **Figure 2** displays the dose distribution in sagittal view, and the second row presents the transversal view of the neck region. As described formerly, the skin region is defined as the first 0.5 cm beneath the outline body, as delineated in red in **Figure 2**. When in the presence of 1.5 T magnetic field, the generated electron streams flow along the magnetic field and then deposit energy on the neck and chin skin areas, as shown in **Figures 2** **A1–A2**. Out-of-field 10% dose region (relative to the prescription dose 4,400 cGy) can spread up to the neck skin, while the 5% dose can stretch to the chin area. However, this phenomenon is totally absent for the plan without magnetic field (as in **B1–B2**), where the scattered in-air electrons show a fairly diffused distribution, with scarce electrons striking the neck and chin region.

**Figure 3:**
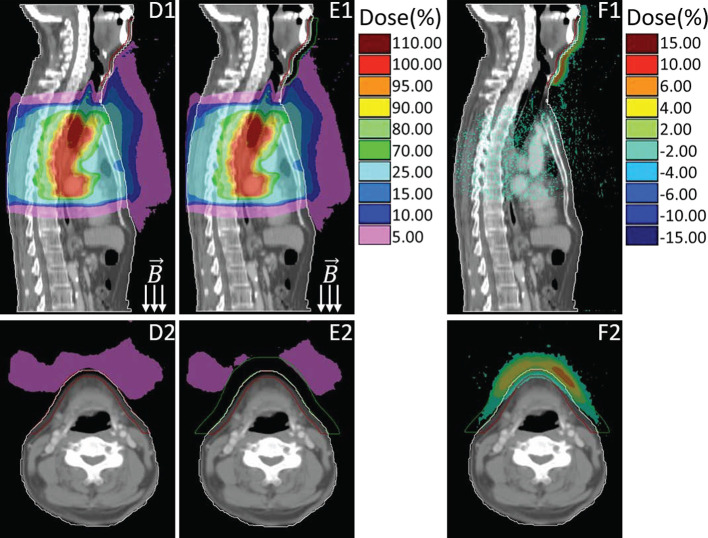
Sagittal and transversal views of the dose distributions for primary plan **(D)**, plan with using bolus **(E)** and the difference (**F**, primary plan–plan with bolus). Colors: Green line delineates the 1cm bolus, other colors are consistent with that in **Figure 2**.

**Figure 3** presented the dose comparison between the primary plan (**D1–D2**) and the plan by using 1 cm virtual bolus (**E1–E2**) for the same representative case. Similarly, their dose differences are also provided (**F1–F2**), which is derived from the primary plan subtracting the plan with bolus. As shown in **Figure 3**, with the shielding of bolus, most of the in-air electron streams will be absorbed before reaching the neck and chin area. The out-of-field 5% dose region induced by ESE can be totally stopped by the bolus, thus protecting the neck and chin skin.

**Figure 4:**
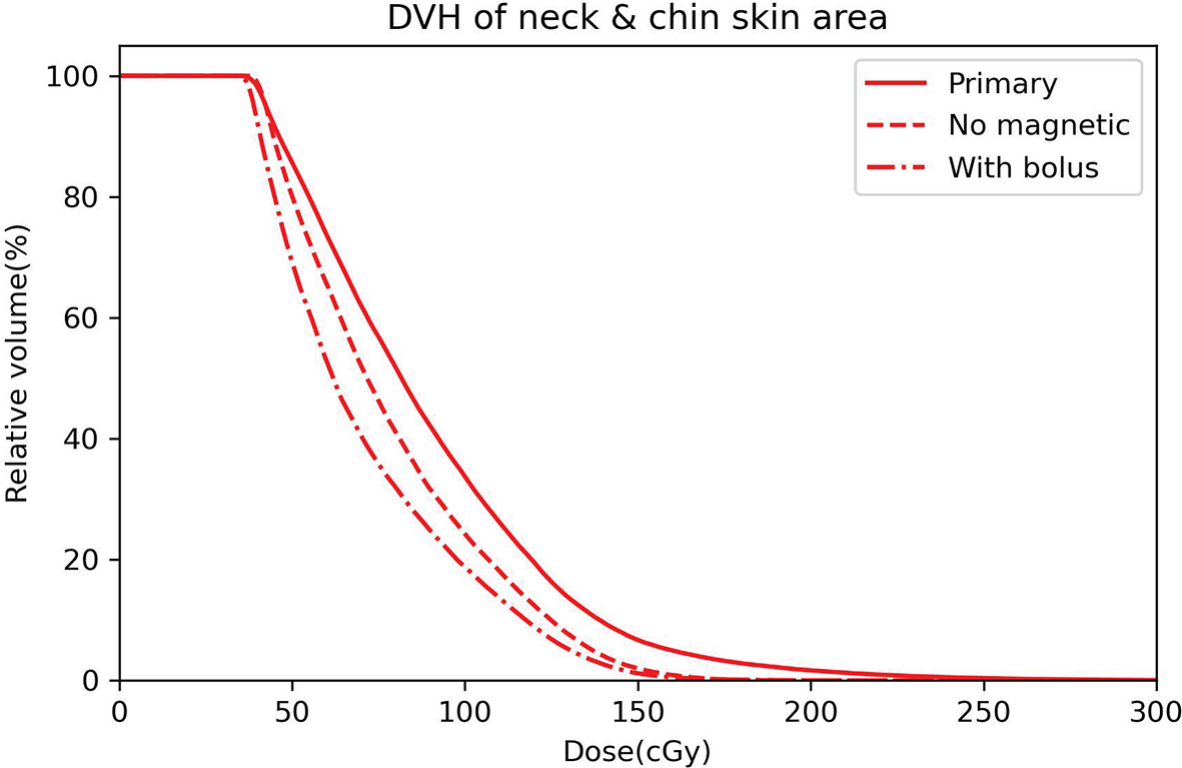
DVH curves of the neck and chin skin area for the primary plan (solid line), plan with no magnetic field (dashed line) and plan with bolus (dot dashed line).

To quantify the dose variation induced by ESE, DVH comparisons of the neck and chin skin region between the three plans (primary, no magnetic field, with bolus) were performed, as shown in **Figure 4**. Significant differences between the DVH curves are observed, especially for the high dose received. The maximum neck and chin skin dose of primary plan can be up to 300 cGy, much higher than that of plan with no magnetic field and plan with bolus (less than 200 cGy). For this representative case, DVH parameters including D_1cc_ (the minimum dose to 1 cc volume), D_2cc_ and D_5cc_ of the neck and chin skin region were also evaluated. The values of these DVH metrics of the three plans are as follows: primary plan: D_1cc_ = 225.5 cGy, D_2cc_ = 201.8 cGy, D_5cc_ = 167.5 cGy; plan with no magnetic field: D_1cc_ = 161.1 cGy, D_2cc_ = 152.9 cGy, D_5cc_ = 140.2 cGy; plan with bolus: D_1cc_ = 154.7, D_2cc_ = 146.4 cGy, D_5cc_ = 134.1 cGy. Significant difference was observed. Meanwhile as shown in **Figure 4**, the overall chin skin dose of plan with using bolus is even lower than the plan with no magnetic field. This indicates that adding a 1 cm bolus can effectively shield the ESE dose.

**Table 2** outlined the statistical results of the DVH metrics for the patient cohort. Wilcoxon singed rank tests were performed to compare those DVH parameters. The mean value coupled with standard deviation error was presented. According to the statistics, the average values of D_max_, D_1cc_, D_2cc_, and D_5cc_ of the primary plan are obviously higher than those of plan without magnetic field (all at p < 0.05), also meanwhile higher than those of plan using bolus (all with p < 0.05), which means that there are significant difference of neck and chin skin doses between the primary plan and the other two plans. Also, adding a 1 cm bolus can significantly mitigate the impact of ESE on neck and chin skin dose. As to the plan with bolus *versus* plan with no magnetic field, no statistically significant differences of these metrics were observed (all with p > 0.05). This indicates that adding the bolus can achieve the shielding effect even equivalent to the scenario with no magnetic field.

**Table 2:** Neck and chin skin area DVH metrics comparisons and statistical analysis for the patient cohort.

	Primary	NoMag^#^	Bolus	p values		
				NoMag vs Primary	Bolus *vs* Primary	Bolus *vs* NoMag
D_max_	657.92 ± 69.07	281.78 ± 36.59	398.43 ± 69.19	0.005	0.007	0.074
D_1cc_	382.06 ± 44.14	214.45 ± 23.82	212.42 ± 23.65	0.005	0.005	0.386
D_2cc_	330.62 ± 39.1	200.71 ± 20.83	193.88 ± 20.05	0.005	0.005	0.241
D_5cc_	259.94 ± 31.49	178.93 ± 31.94	169.55 ± 15.82	0.005	0.005	0.059

# NoMag represents the plan with no magnetic field.

## Discussion

Esophageal cancer patients are a potential group to benefit from MRL owing to the better image guidance with high soft-tissue contrast. However, due to the presence of 1.5 T perpendicular magnetic field during treatment delivery, scattered in-air secondary electrons can be compelled to spiral along the magnetic field line (in-air ESE), leading to the variation of out-of-field doses. In our study of thoracic esophageal cancer treatment planning under 1.5 T perpendicular magnetic field, out-of-field in-air ESE was also observed, leading to an increase to the neck and chin skin dose when compared with no magnetic field. To our knowledge, the present study is the first to investigate the ESE impact on out-of-field doses of the esophageal cancer treatment planning under magnetic field. Scattered or backscattered in-air electrons generated by the photon beams interacting with materials contributed to these electron streams. The streams can travel along the magnetic field lines for a long distance and potentially strike the patient’s body outside the treatment field, thus contribute to the skin toxicity. For esophageal cancer cases, the in-air streaming electrons are mainly direct toward the neck and chin skin area, but also a small portion can drift along the feet direction, as shown in **Figsure 2 A1**. This is due to the irregular interface between the body contour and air, streaming electrons toward the feet direction are easily stopped by the nearby in-field bulge region of body surface. In contrast, the electrons drifted toward the cranial are unobstructed, thereby could deposit energy to the remote protruding regions, such as neck and chin areas.

ESE is a special phenomenon observed on MRL system, but also ubiquitously existed when in the presence of magnetic field. Previous case studies performed either on Unity or ViewRay system mainly focused on the ESE during MRI guided breast cancer treatment (20, 21, 25). However, in breast case planning, the photon beams are typically set tangential to the tumor site, thus undergo a greater attenuation along the beam path when compared with the esophageal case. According to the water phantom based Monte Carlo simulation performed by Molkov et al, thickness of the treatment region does play a role in the magnitude of ESE doses (18). Thicker irradiation region comes with greater photon attenuation, thus fewer in-air electrons will be produced and focused by the magnetic field (18), which means that thinner or less attenuating regions (*e.g.* esophageal cancer) can probably result in a relatively higher ESE doses. In our present work, it is indicated that the skin dose escalation induced by ESE for esophageal cancer is also significant, even severer than for breast cancer. For example, in one extreme case of our study, the maximum chin skin dose is 25.2% of the prescription dose, which is higher than the extreme breast case reported by Park et al., of which the corresponding maximum dose to the patient’s chin skin surface is 16.1% of the prescription dose (20). On average, the maximum chin skin dose induced by ESE of the patient cohort reported by Park et al. is 5.4% of the prescription, whereas in our study for esophageal cancer, the average chin skin maximum dose is 15.0% of the prescription dose. Another single case study of breast cancer conducted on Unity MRL system by Nachbar et al. found that the presence of 1.5 T magnetic field can induce as large as 6.5% of the prescription dose to the chin area during the partial breast irradiation (21). Noted that the chin dose in their study has an average area of 6.0 cm^2^. That is comparable to our result of metric D_5cc_. For the extreme case in our study, D_5cc_ of the chin skin is 9.6% of the prescription dose, higher than that reported by Nachbar et al. On average, D_5cc_ of the chin and neck skin dose for the esophageal patient cohort in our study is 5.9% of the prescription dose. According to the data in **Table 2**, large difference between the metric D_max_ and D_1cc_ of primary plan is observed, which means that only small number of voxels in the skin area is deposited with relatively extremely high ESE dose. Considering the statistical uncertainty of Monte Carlo dose calculation, evaluation using D_1cc_, D_2cc_, and D_5cc_ should make more sense.

The question of how to handle with the ESE induced skin dose arises can be resolved by adding the bolus, as indicated by previous studies about MRI guided breast irradiation (20). Further simulation studies also demonstrated that out-of-field streaming electrons can only penetrate with few millimeters in the material (18). Considering the electron streams generated in Unity system have relatively low energies and therefore short ranges (18). Shielding effect of virtually adding a 1 cm bolus was also investigated in our study. If without using a bolus, the streaming electrons would deposit energy and lead to unwanted dose to the neck and chin skin areas. In an extreme case, the D_1cc_ of out-of-field chin skin area can be as high as 636.4 cGy, nearly 14.5% of the prescription. This is because the PTV upper boundary in that case is much close to the chin (with the minimum distance of 2.7 cm from PTV upper border to the skin started), and the strength of ESE is relevant to that distance. But if using the bolus, the corresponding skin dose D_1cc_ can be much lower, approximately 7.4% of the prescription dose, even lower than the case without magnetic field (with D_1cc_ of 8.5%). For the patient cohort, on average the metric D_1cc_ can be reduced to 4.8% of the prescription when using the bolus, compared with the corresponding D_1cc_ value of 8.7% for primary plans without bolus. Which means that adding a 1 cm bolus can also effectively mitigate the ESE impact for MRI guided esophageal cancer, doses to the neck and chin skin surface can be reduced. However, ESE does not only exist in the air outside the patient’s body, but also can occur inside, as long as in the presence of air cavity. As presented in **Figure 2**, prominent ESE is also observed in the upper trachea region inside the patient’s body. Scattered electrons can be converged by magnetic field to form streams in the trachea air cavity and drift along the magnetic field line, thus may lead to a high surface dose in trachea wall. The electron streams formed outside the body can be effectively shielded by adding bolus, but the ESE that occurred inside the body’s air cavity is merely impossible to resolve. However, the inside body ESE induced doses for out-of-field trachea wall regions are lower than 25% of the prescription dose and also limited in a small influenced area (**Figure 2** **A1**). Considering the dose limit of trachea (<30 Gy) for esophageal cancer radiotherapy (26), the impact due to ESE is not likely to cause toxicity.

This study only investigated the impact of ESE for esophageal cancer treatment planning with the same prescription dose and same beam angles. Whereas the magnitude of generated electron streams is relevant to the gantry angle and incline of the patient body surface, as indicated by Malkov et al (18). Electron streams formed at the beam exit side and meanwhile at inclined exit surface will pose a stronger magnitude (18). This may provide a guidance on how to mitigate the ESE by adjusting the gantry angles, and it will be investigated in future work using treatment planning and also measurements. In addition, the data and conclusion derived from the retrospective study with ten patients and identical prescription dose are limited. The extent and potential impact of ESE need to be validated in a larger cohort of patients to incorporate various situations, such as higher prescription and extreme target location. Nevertheless, our findings indicate that ESE for esophageal cancer should be taken into account at the planning phase, and the induced unwanted *in-vitro* doses deposited outside the treatment field could be reduced. Suggestions such as including the chin region in planning images of the patient, displaying the out-of-field low doses to the neck and chin area in TPS, delineating the skin region as OAR for evaluation, even optimizing the plan if a much higher dose to the skin was observed and placing a bolus on the neck and chin during treatment delivery could be considered. Another limitation of this study is that the TPS calculated out-of-field dose distributions with or without the bolus has not been validated by the measured doses, which is a top priority work we are still undertaking. To verify the coincidence between the calculated and measured out-of-field dose distributions caused by ESE in magnetic field, an anthropomorphic phantom rather than actual patients should be more appropriate considering the better positioning accuracy and reproducibility.

## Conclusion

Out-of-field dose escalation induced by ESE is also a significant issue for MRI guided esophageal cancer radiotherapy, even more protruding than previous studies about breast cases. A high out-of-field neck and chin skin doses were observed in the presence of magnetic field, and this should be considered in the treatment planning. This phenomenon was intensified when tumor sites were located in the upper thorax. While for conventional radiotherapy in the absence of magnetic field, there is no need to consider those scattered in-air electrons. Adding 1 cm bolus on top of the patient’s neck and chin region could effectively reduce the ESE dose contributions, achieve the shielding effect almost equivalent to the scenario with no magnetic field. Further explorations of measurement verifications for the Monaco system calculated out-of-field ESE dose distributions are required.

## Data Availability Statement

The original contributions presented in the study are included in the article/supplementary material. Further inquiries can be directed to the corresponding authors.

## Ethics Statement

The studies involving human participants were reviewed and approved by Sun Yat-sen University Cancer Center institutional review board (IRB, C2019-006-01). Written informed consent for participation was not required for this study in accordance with the national legislation and the institutional requirements.

## Author Contributions

All authors contributed to the article and approved the submitted version.YS and XH participated in the conceptualization of this study. HL and XH contributed to the study design and drafted the manuscript. SD and HL collected the planning data and performed the replanning workflow. BW and YL participated in the data analysis.

## Funding

This work was supported by National Natural Science Foundation of China (No. 11805292); Natural Science Foundation of Guangdong, China (No. 2018A0303100020).

## Conflict of Interest

The authors declare that the research was conducted in the absence of any commercial or financial relationships that could be construed as a potential conflict of interest.

## References

[1] Bray F Ferlay J Soerjomataram I Siegel R Torreet L Jemal A Global cancer statistics 2018: GLOBOCAN estimates of incidence and mortality worldwide for 36 cancers in 185 countries. CA: Cancer J Clin (2018) 68(6):394–424. 10.3322/caac.21492 30207593

[2] Ferlay J Soerjomataram I Dikshit R Eser S Mathers C Rebelo M Cancer incidence and mortality worldwide: sources, methods and major patterns in GLOBOCAN 2012. Int J Cancer (2015) 136(5):E359–86. 10.1002/ijc.29210 25220842

[3] Voncken F Nakhaee S Stam B Wiersema L Vollenbrock S van Dieren J Quantification of esophageal tumor motion and investigation of different image-guided correction strategies. Pract Radiat Oncol (2020) 10(2):84–92. 10.1016/j.prro.2019.11.012 31786235

[4] Slotman B Gani C Online MR-guided radiotherapy–A new era in radiotherapy. Clin Trans Radiat Oncol (2019) 18:102–3. 10.1016/j.ctro.2019.04.011 PMC6630179 31341984

[5] Winkel D Bol G Kroon P van Asselen B Hackett S Werensteijn-Honingh A Adaptive radiotherapy: the Elekta Unity MR-linac concept. Clin Trans Radiat Oncol (2019) 18:54–9. 10.1016/j.ctro.2019.04.001 PMC6630157 31341976

[6] Nachbar M Mönnich D Kalwa P Zips D Thorwarth D Gani C Comparison of treatment plans for a high-field MRI-linac and a conventional linac for esophageal cancer. Strahlentherapie und Onkologie (2019) 195(4):327–34. 10.1007/s00066-018-1386-z 30361744

[7] Boekhoff M Defize I Borggreve A Takahashi N van Lier A Ruurda J 3-Dimensional target coverage assessment for MRI guided esophageal cancer radiotherapy. Radiother Oncol (2020) 147:1–7. 10.1016/j.radonc.2020.03.007 32234611

[8] Menten M Fast M Nill S Kamerling C McDonald F Oelfke U Lung stereotactic body radiotherapy with an MR-linac–Quantifying the impact of the magnetic field and real-time tumor tracking. Radiother Oncol (2016) 119(3):461–6. 10.1016/j.radonc.2016.04.019 PMC4936791 27165615

[9] Glitzner M Woodhead P Borman PT Lagendijk J Raaymakers B MLC-tracking performance on the Elekta unity MRI-linac. Phys Med Biol (2019) 64(15):15NT02. 10.1088/1361-6560/ab2667 31158831

[10] Stemkens B Glitzner M Kontaxis C de Senneville B Prins F Crijns S Effect of intra-fraction motion on the accumulated dose for freebreathing MR-guided stereotactic body radiation therapy of renal-cell carcinoma. Phys Med Biol (2017) 62:7407–24. 10.1088/1361-6560/aa83f7 28771144

[11] Kontaxis C Bol G Kerkmeijer L Lagendijk J Raaymakers B Fast online replanning for interfraction rotation correction in prostate radiotherapy. Med Phys (2017) 44(10):5034–42. 10.1002/mp.12467 28703497

[12] Kontaxis C Bol G Stemkens B Glitzner M Prins F Kerkmeijer L Towards fast online intrafraction replanning for free-breathing stereotactic body radiation therapy with the MR-linac. Phys Med Biol (2017) 62(18):7233. 10.1088/1361-6560/aa82ae 28749375

[13] Woodings S Bluemink J De Vries J Niatsetski Y van Veelen B Schillings B Beam characterisation of the 1.5 T MRI-linac. Phys Med Biol (2018) 63(8):085015. 10.1088/1361-6560/aab566 29521280

[14] Raaymakers B Jürgenliemk-Schulz I Bol G Glitzner M Kotte A van Asselen B First patients treated with a 1.5 T MRI-Linac: clinical proof of concept of a high-precision, high-field MRI guided radiotherapy treatment. Phys Med Biol (2017) 62(23):L41. 10.1088/1361-6560/aa9517 29135471

[15] Hissoiny S Raaijmakers A Ozell B Després P Raaymakers B Fast dose calculation in magnetic fields with GPUMCD. Phys Med Biol (2011) 56(16):5119. 10.1088/0031-9155/56/16/003 21775790

[16] Hackett S van Asselen B Wolthaus J Bluemink JJ Ishakoglu K Kok J Spiraling contaminant electrons increase doses to surfaces outside the photon beam of an MRI-linac with a perpendicular magnetic field. Phys Med Biol (2018) 63(9):095001. 10.1088/1361-6560/aaba8f 29595150

[17] Malkov V Hackett S van Asselen B van Asselen B Wolthaus J Monte Carlo simulations of out-of-field skin dose due to spiralling contaminant electrons in a perpendicular magnetic field. Med Phys (2019) 46(3):1467–77. 10.1002/mp.13392 PMC6850151 30666678

[18] Malkov V Hackett S Wolthaus J Raaymakers B Van Asselen B Monte Carlo simulations of out-of-field surface doses due to the electron streaming effect in orthogonal magnetic fields. Phys Med Biol (2019) 64(11):115029. 10.1088/1361-6560/ab0aa0 30808017

[19] An H Kim J Park J Electron streams in air during magnetic-resonance image-guided radiation therapy. PLoS One (2019) 14(5):e0216965. 10.1371/journal.pone.0216965 31091270 PMC6519819

[20] Park J Shin K Kim J Park S Jeon S Choi N Air–electron stream interactions during magnetic resonance IGRT. Strahlentherapie und Onkologie (2018) 194(1):50–9. 10.1007/s00066-017-1212-z 28916952

[21] Nachbar M Mönnich D Boeke S Gani C Weidner N Heinrich V Partial breast irradiation with the 1.5 T MR-Linac: First patient treatment and analysis of electron return and stream effects. Radiother Oncol (2020) 145:30–5. 10.1016/j.radonc.2019.11.025 31874347

[22] Ng-Cheng-Hin B Nutting C Newbold K Bhide S McQuaid D Dunlop A The impact of restricted length of treatment field and anthropometric factors on selection of head and neck cancer patients for treatment on the MR-Linac. Br J Radiol (2020) 93:20200023. 10.1259/bjr.20200023 32436787 PMC7336067

[23] Chuter R Whitehurst P Choudhury A van Herk M McWilliam A Investigating the impact of field size on patient selection for the 1.5 T MR-Linac. Med Phys (2017) 44(11):5667–71. 10.1002/mp.12557 28869651

[24] Bol G Lagendijk J Raaymakers B Virtual couch shift (VCS): accounting for patient translation and rotation by online IMRT re-optimization. Phys Med Biol (2013) 58(9):2989. 10.1088/0031-9155/58/9/2989 23588253

[25] Koerkamp M Vasmel J Russell N Shaitelman S Anandadas C Currey A Optimizing MR-Guided Radiotherapy for Breast Cancer Patients. Front Oncol (2020) 10:1107. 10.3389/fonc.2020.01107 32850318 PMC7399349

[26] Ritter T Quint DJ Senan S Consideration of dose limits for organs at risk of thoracic radiotherapy: atlas for lung, proximal bronchial tree, esophagus, spinal cord, ribs, and brachial plexus. Int J Radiat Oncol Biol Phys (2011) 81(5):1442–57. 10.1016/j.ijrobp.2010.07.1977 PMC3933280 20934273

